# 2197. Genomic Surveillance for Human Adenovirus Infections among Department of Defense Beneficiaries in the 2018-2019 Season

**DOI:** 10.1093/ofid/ofac492.1816

**Published:** 2022-12-15

**Authors:** Adam Pollio, Anthony C Fries, Yu Yang, Jerry Hughes, Christian Fung, Matthew Conte, Natalie Collins, Elizabeth A Macias, Jun Hang

**Affiliations:** Walter Reed Army Institute of Research, Silver Spring, Maryland; U.S. Air Force School of Aerospace Medicine, Dayton, Ohio; USDA, Beltsville, Maryland; Walter Reed Army Institute of Research, Silver Spring, Maryland; Walter Reed Army Institute of Research, Silver Spring, Maryland; Walter Reed Army Institute of Research, Silver Spring, Maryland; Walter Reed Army Institute of Research, Silver Spring, Maryland; U.S. Air Force School of Aerospace Medicine Epidemiology Laboratory Service, Wright-Patterson AFB, Ohio; Walter Reed Army Institute of Research, Silver Spring, Maryland

## Abstract

**Background:**

Human adenoviruses (family *Adenoviridae*, genus *Mastadenovirus*) (HAdV) species B, C and E cause acute respiratory illnesses (ARI). In particular, respiratory HAdV types 3, 4, 7, 11, 14, 21 and 55 were associated with ARI outbreaks in the US and in other countries. The risk of outbreaks can be effectively controlled by implementation of the live HAdV types 4 and 7 vaccines or mitigated by screening and preventive measures.

**Methods:**

During the influenza season 2018 – 2019, the DoD Global Respiratory Pathogen Surveillance Program (DoDGRS) received 24,300 specimens from its surveillance network. In total, 322 (1.3%) respiratory specimens were positive for HAdV in molecular assays. HAdV samples that produced positive cytopathic effects (CPE) in subsequent viral cultivation were subjected to next-gen sequencing (NGS) for genome sequence assembly, HAdV genome typing, whole genome phylogeny, and sequence comparative analyses to identify sequence insertions, deletions and amino acid mutations.

**Results:**

Out of 166 viral culture samples available for NGS, whole genome sequences were obtained for 161 isolates. A variety types of HAdV were identified, including HAdV-1 (N = 15), HAdV-2 (N = 26), HAdV-3 (N = 60), HAdV-4 (N = 13), HAdV-5 (N = 6), HAdV-6 (N = 3), HAdV-7 (N = 12), HAdV-14 (N = 10), HAdV-55 (N = 1), and HAdV-56 (N = 1). HAdV types 4, 7 and 14, which were reported causing ARI outbreaks in the US military and other congregate settings, were found in clustered cases in several US states. Comparative sequence analyses of these isolates revealed the emergence of novel genetic mutations despite the stability of adenovirus genomes.

**Conclusion:**

Routine clinical respiratory pathogen diagnostics or molecular surveillance of respiratory diseases detect adenovirus without determination of HAdV type. Human adenoviruses are genetically diverse and can infect a number of tissues with severities varied from mild to fatal. Enhanced genomic surveillance of HAdV in the US and worldwide will shed light on prevalence, genetic divergence, and viral evolution of human adenoviruses and inform timely risk assessment and countermeasures.

**Disclosures:**

**All Authors**: No reported disclosures.

